# The association between dietary patterns and quality of life: a cross-sectional study among a large sample of industrial employees

**DOI:** 10.1186/s12889-023-16898-9

**Published:** 2023-10-17

**Authors:** Saeede Jafari Nasab, Sahar Golpour Hamedani, Hamidreza Roohafza, Awat Feizi, Cain C. T. Clark, Nizal Sarrafzadegan

**Affiliations:** 1 Department of Clinical Nutrition, School of Nutrition and Food Sciences, Food Security Research Center, Isfahan University of Medical Sciences, Isfahan, Iran; 2 Department of Community Nutrition, School of Nutrition and Food Science, Food Security Research Center, Isfahan University of Medical Sciences, Isfahan, Iran; 3 Cardiac Rehabilitation Research Center, Cardiovascular Research Institute, Isfahan University of Medical Sciences, Isfahan, Iran; 4 Department of Biostatistics and Epidemiology, School of Health, Isfahan University of Medical Sciences, HezarJarib Ave, P.O. Box 319, Isfahan, Iran; 5 Centre for Intelligent Healthcare, Coventry University, Coventry, CV1 5FB UK; 6 Isfahan Cardiovascular Research Center, Cardiovascular Research Institute, Isfahan University of Medical Sciences, Isfahan, Iran

**Keywords:** Quality of life, Dietary pattern, Industrial workers, Manufacturing employees

## Abstract

**Background:**

considering the diet, as a whole (dietary patterns), can provide more information regarding dietary guidelines to decrease health problems and improve quality of life (QoL) of industrial workers.Therefore, the aims of this study were to identify major dietary patterns and to evaluate their association with quality of life among Iranian industrial employees.

**Methods:**

This cross-sectional study was conducted on 3,063 employees of Isfahan Steel Company, Isfahan, Iran, in 2015. Dietary data were evaluated through a validated form of a food frequency questionnaire. Exploratory factor analysis was used to extract major dietary patterns,. To assess the QoL, Euro-QoL five- dimension questionnaire was used. Latent class analysis was used to classify participants based on QoL. Multivariable logistic regression was employed to evaluate the association between dietary patterns and QoL.

**Results:**

Three dietary patterns, i.e. western, healthy and traditional, and two classes, i.e. high and low quality of life. were identified from study participants. Lower adherence to the healthy dietary pattern increased the risk of being in low QoL class in which subjects in the lowest tertile of healthy dietary intake had higher odds of being in low QoL class (adjusted OR (AOR): 1.51, 95% CI: 1.19–1.91). However, subjects in the lowest tertile of traditional diet, low adherence, had 30% lower risk of belonging to the low QoL class (AOR:0.70, 95% CI: 0.55–0.88). Higher adherence to western dietary pattern increased the risk of low quality of life, but it was not statistically significant.

**Conclusion:**

Higher adherence to a healthy diet and lower adherence to traditional dietary pattern were associated with better QoL in manufacturing employees.

**Supplementary Information:**

The online version contains supplementary material available at 10.1186/s12889-023-16898-9.

## Background

According to the World Health Organization (WHO), quality of life (QoL) is defined as “An individual’s perception of their position in life in the context of the culture and value systems in which their lives in relation to their goals, expectations, standards and concerns” [1]. QoL can be judged either in an objective or subjective manner, and also it includes physical, environmental, psychological, and social aspects of life [2, 3].

Quality of work or productivity, which is reflected in absenteeism, presenteeism, productivity, and work performance, can be affected by the QoL [4, 5]. The association of several factors, such as age, gender, educational level, sleep hours, fatigue, medical condition, psychosocial status, physical activity, body mass index (BMI), smoking, and diet with QoL has been studied [4, 6, 7]. Some studies have reported that the adequate nutrition of workers is crucial for better production levels. Studies in this field have primarily focused on the assessment of nutritional status and reported the intake of energy, vitamins and minerals [8, 9]. Insufficient and imbalanced nutrition poses a significant health concern, especially among the workforce that could reduce both work capacity and resistance to diseases and increase job-accidents [10].

Recently, an interest in the impact of diet on QoL in health and diseases has increased. For instance, the association of single nutrients, food groups, and dietary interventions with QoL for several diseases, such as cancers, diabetes, irritable bowel syndrome, cardiovascular diseases, and obesity, has been explored [11–17]. However, data regarding the working population, especially in industrial workers, are sparse [18, 19].

Although investigating the effects of single nutrients and foods on health and diseases has enhanced our knowledge of the fundamental biological pathways involved in how diet influences body, considering the impact of diet, as a whole (dietary patterns), can provide more information regarding the appropriate dietary guidelines to decrease health problems, improve QoL of industrial workers, and reduce economic burden on their families, especially in developing nations [20].

Dietary patterns can be defined by two approaches: a priori and a posteriori. In the priori approach, the scores of compliance to predefined diets are calculated (e.g., Healthy Eating Index, Mediterranean diet score and Dietary Approaches to Stop Hypertension), while in the a posteriori method, patterns can be driven using principal component analysis (PCA) or cluster analysis [21].

To the best of our knowledge, only a few studies have been conducted to investigate an association between dietary patterns and QoL among industrial workers.. Considering that dietary patterns can provide important information about lifestyle of individuals as well as QoL and its effects on productivity and other aspects of QoL; therefore, we aimed to identify major dietary patterns and to evaluate their association with QoL among industrial employees.

## Materials and methods

### Study design and population

This cross-sectional study was conducted in 2015 on 3500 formal and contractual employees working in Isfahan Steel Company, Isfahan, Iran. The required sample size was determined to be 3500, considering the prevalence of the mental health problems as 0.1, type one error rate as 0.05, and a sampling error rate of 0.01 [22, 23]. Our study sample was selected among 16,000 people using both multi-stage cluster sampling and stratified sampling. Clusters consisted of seven major management departments and their subordinate sections and job categories of employees were considered as strata. The sample sizes for clusters and strata were proportional to the size of each department. Participants were eligible to enroll in this study if they had at least one year of work experience and agreed to participate in the study. Moreover, who did not answer more than 10% of questions (*n* = 437 employees) were excluded from the study. Considering that the number of female employees in each department was low, only 260 women, who were willing to participate in the study, all were included using the convenience sampling. Finally 3063 people with complete data have been subjected to data analysis. Prior to thier participation in the study, all subjects provided the written informed consent. More details about study structure and content have been presented previously [22]. We followed the ‘Strengthening the Reporting of Observational Studies in Epidemiology’ (STROBE) statement.

### Dietary assessment

Usual dietary intake of the participants was assessed via a validated self-administered food frequency questionnaire (FFQ-short form) [24]. If participants answered “yes” to their consumption of a specific food, they were asked to define their frequency of consumption for each item on a daily, weekly, or monthly basis. Afterwards, the frequencies of intakes were converted to times of consumption per week. Intake classified as seldom and never was determined as “zero”. The score of each dietary pattern extracted by factor analysis was considered as predictor in regression models.

### Quality of life

In this study, one of the most widely used generic instruments for assessing QoL, i.e., Euro QoL-five- dimension questionnaire (EQ-5D-3L), was utilized [25]. This is a two-part self-report instrument. The first part (self- classifier) has 5 generic domains, including mobility, self-care, normal activities, pain/discomfort, anxiety, and depression. Each domain consists of three levels, by which participants describe their health state: no problem, some problems, sever problems. Higher EQ-5D scores indicate worse health status. The second part is the visual analog scale (VAS); this is a scale ranging from 0 (worst imaginable health state) to 100 (best imaginable health state). The participants were asked to rate their current health state by drawing a single vertical line on the VAS. In the current study, the Persian version of EQ-5D, which is a valid and reliable questionnaire, was used [5, 26]. The two categories of “some” and “severe” were merged into a single category in the analysis due to low response rate in the severe category. Higher EQ-5D scores show poor QoL. The QoL and extracted classes based on using latent class analysis were considered as response variables in regression models.

### Covariate assessment

Necessary information about the socio-demographic variables (such as sex, age, educationlevel, marital status, and household size), job-related variables (such as shift work, having second job and job stress), and lifestyle variables (such as sleep duration, body mass index and smoking) were collected by the validated structured and self-administered questionnaires. Physical activity was evaluated using the short form of the International Physical Activity Questionnaire (IPAQ), which is a valid tool to assess physical activity in the Iranian population [27]. In this questionnaire, participants answered questions about frequency and duration of activities. Corresponding activities were divided into vigorous intensity, moderate intensity, and walking and sitting scores. A total measure of physical activity was calculated from summation of all three scores. All variables, which are self-reported, may have recall bias or over and under stated.

### Statistical analysis

To extract the major dietary pattern, Exploratory Factor Analysis (EFA), based on Principal Component Analysis (PCA) extraction method, was performed on 38 food items and food groups. The orthogonal Varimax rotation procedure was used to find the interpretable factors. The number of factors to retain was guided by scree plot inspection and eigenvalues > 1. Post-rotated factor loadings showed that three interpretable dietary patterns were labeled based on food groups with the highest loading in each factor. For each dietary pattern, factor score was calculated by summing the intake of food items weighted by their factor loadings, and also a score for each identified pattern was allocated to each person. The participants were categorized based on tertiles of dietary pattern scores. This enabled us to evaluate the association of different levels of intakes from each dietary pattern with QoL in our study participants.

Latent class analysis (LCA) was used to determine latent classes of employees based on their response to 5 domains of the EQ-5D. The modeling process was fitted as follows: in the first step, to classify the subjects according to their response to five items of QoL, LCA was used and LCA with different classes was fitted to define the optimal number of latent classes, and also the number of classes was increased from 1 to 5, successively. To attain the best fitting model, Bayesian information criterion (BIC) and the Lo–Mendell–Rubin likelihood ratio test were used [28, 29]. During the model fitting,the lowest BIC, and theinterpretability of classes were considered concurrently. A two-level model with two classes (level 1 and level 2) at the second level was chosen as the best fit [5].

Binary logistic regression was used to evaluate the association of dietary patterns as the independent variables in the crude model, and adjusted by age and sex (model 1), and age, sex, shifting work, smoking status, BMI, and sleep duration (model 2), with the extracted classes of QoL as the response variable. The last tertile of dietary pattern scores was considered as the reference category in the analysis. We also evaluated the association of each dietary pattern with the score of QoL assessed by VAS by using Pearson correlation and linear regression. Additionally, we compared mean score of each dietary pattern between people suffering and not suffering from each five problems evaluated by EQ-5D questionnaire by independent samples t-test and analysis of covariance after the adjustment for potential confounders.

Continuous and categorical variables were expressed as mean ± SD and number (percentage), respectively. Independent samples t-test for normally distributed variables and Mann–Whitney U test for non-normal quantitative variables, respectively, were used and also chi-square test was used to compare continuous and categorical variables in the study groups. One-way analysis of variance (ANOVA) and the chi-square test were applied to compare means of continuous and categorical variables across tertiles of dietary pattern scores, respectively. All statistical analyses were performed using SPSS version 16(SPSS Inc., Chicago, IL, USA), and also *P* < 0.05 was considered atasitically significant.

## Results

**Fig. 1:**
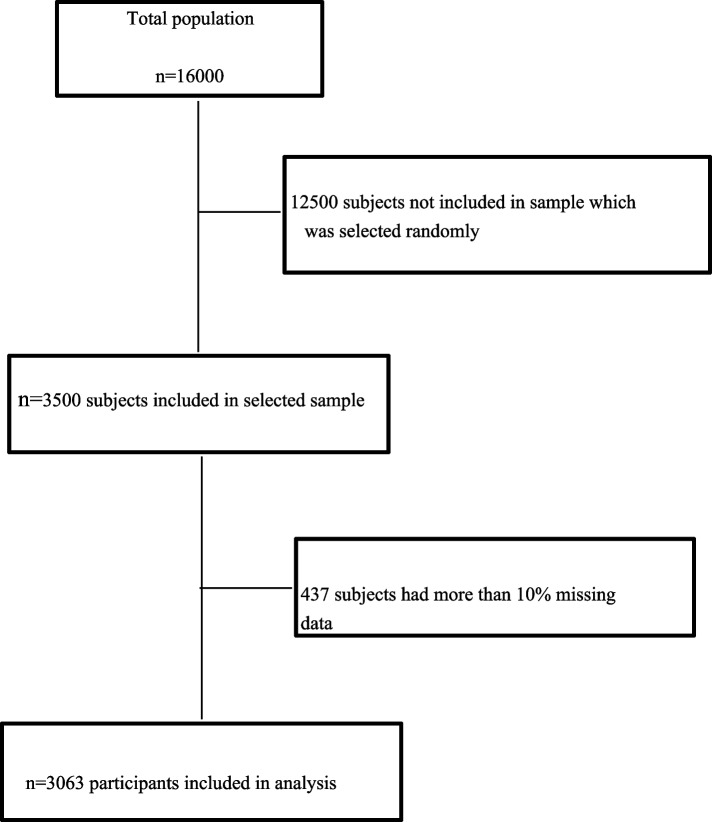
Flow diagram of study samples selection and analysis

A total of 3,063 participants who completed the questionnaires were included in the current study (Fig. 1).

Table 1 presents the two identified classes of participants by applying latent class analysis (LCA) on five domains of EQ-5D questionnaire. Interpretation and the labeling of extracted classes are based on the frequency of picking of “no problem” or “some/severe problems” in all five dimensions by the study participants. As can be seen in Table 1, the probability of picking of “no problem” option for all five dimensions of EQ-5D questionnaire for subjects in second class was very high. Accordingly, this class was considered as high QoL and contained majority of study participants (80%). Moreover, the first class included the subjects with higher frequency of declaring “some/severe problems” in all dimensions of EQ-5D questionnaire; therefore, it was labeled as low QOL and also 20% of the samples were classified in this calss [5].

**Table 1:** Latent classes of quality of life identified by latent class analysis

Items of EQ-5D questionnaire	Levels of quality of life (Class size)	
	Low 621(0.20)	High 2442(0.80)
**Mobility**		
I have no problems in walking about	0.69	0.99
I have some problems in walking about/I am confined to bed	0.31	0.01
**Self-care**		
I have no problems with self-care	0.99	1.00
I have some problems washing or dressing myself/I unable to wash or dress myself Usual activities (e.g. work, study, housework, family or leisure activities)	0.01	0.00
**Daily activity**		
I have no problems with performing my usual activities	0.95	1.00
I have some problems with performing my usual activities/I am unable to perform my usual activities	0.05	0.00
**Pain/discomfort**		
I have no pain or discomfort	0.94	1.00
I have moderate pain or discomfort/I have extreme pain or discomfort	0.06	0.00
**Anxiety/depression**		
I am not anxious or depressed	0.13	0.89
I am moderately anxious or depressed/I am extremely anxious or depressed	0.87	0.11

Data are presented as percentage

Table 2 describes the differences between low and high QoL classes in terms of demographic, job- related, and lifestyle factors. People in the high QoL class were more significantly younger (36.26 ± 7.26 vs. 38.48 ± 7.22), male (92.4% vs. 88.1%), non-smokers (71.9% vs. 67%), shift worker (56.4% vs. 49.1%), and had lower BMI (25.46 ± 3.70 vs. 26.09 ± 4.11), and higher sleep duration (7.16 ± 1.15 vs. 6.91 ± 1.22) as compared with those in the low QoL class (*P* < 0.05).

**Table 2:** Demographic characteristics, life style, job-relate variables of participants across two classes of QoL

Level of QoL(QoL class)			
Variables	Low	High	*P*-value*
Gender			
Male	547(88.1)	2256(92.4)	0.001
Female	74(11.9)	186 (7.6)	
Marital status			
Married	568(91.5)	2190(89.7)	0.185
Single	53(8.5)	252(10.3)	
Education level			0.193
0–5 years	41(6.6)	214(8.8)	
6–12	389(62.6)	1519(62.2)	
> 12	191 (30.8)	709(29)	
Smoking status			
Non smokers	416(67)	1755(71.9)	0.017
Current or former smoker	205(33)	687(28.1)	
Shift work			0.001
Daily (daily shift)	316(50.9)	1064(43.6)	
Shift (rotating shift work)	305(49.1)	1378(56.4)	
Second job yes	54(8.7)	231(9.5)	0.559
Age (years)	38.48 ± 7.22	36.26 ± 7.26	< 0.001
BMI (Kg/m^2^)	26.09 ± 4.11	25.46 ± 3.70	< 0.001
Physical activity (METs hour/week)	9.20 ± 14.51	9.97 ± 15.55	0.26
Sleep duration (hour)	6.91 ± 1.22	7.16 ± 1.15	< 0.001
Income (Rials)			< 0.001
< 5,000,000	90 (14.5)	391(16)	
5,000,000–8000000	285(45.9)	1301(53.3)	
8,000,000- 12,000,000	164(26.4)	496(20.3)	
> 12,000,000	80(12.9)	246(10.1)	
No answer	2(0.3)	8(0.3)	

^*^P-values resulted from two independent samples t-test (or Mann–Whitney U test) and chi-squared test for continues and categorical variables, respectively

Table 3 shows the dietary patterns extracted using factor analysis. Three major dietary patterns were identified based on 38 food items/groups. First, a ‘western’ dietary pattern was defined by high intake of butter, cream, organ meats, processed meat, spaghetti, carbonated drinks, jams, pastry, biscuits, cookies, chocolates, fresh fruit juice, all kinds of seeds, fruit juice, canned food, fast foods, mayonnaise, and egg, second, a ‘healthy’ dietary pattern was highly loaded with liquid oil, cheese, low-fat milk, low-fat yoghourt, yoghourt-based beverage, fish, fruits, vegetables, nuts, soya, garlic and beans, and third, a ‘traditional’ dietary pattern was characterized by higher intakes of hydrogenated oil, high-fat milk, high-fat yoghourt, red meat, poultry, all kinds of bread, rice, potato, fried foods and restaurant foods; these patterns explained 7.8%, 6.3%, and 6.1% of the total variance in dietary intake, respectively.

**Table 3:** Factor loadings for food items in three extracted factors as dietary patterns

Food items /groups	western	Healthy	Traditional
Butter	0.38		
Cream	0.44		
Liver, heart and kidney	0.44		
Organ meat	0.31		
Proceed meats	0.53		
pasta	0.35		
Carbonated drinks	0.53		
Jams	0.40		
Cakes, cookies and sweets	0.38		
Seeds	0.36		
Industrial fruit juices	0.47		
Egg	0.31		
Canned Foods	0.34		
Fast food	0.55		
Restaurant foods	0.11		
Mayonnaise	0.40		
Non-hydrogenated Vegetable oil		0.24	
Cheese		0.20	
Low fat milk		0.35	
Low fat Yogurt		0.46	
Yogurt-based beverage		0.44	
Fish		0.37	
Fruits		0.43	
Fresh fruit juice		0.24	
Vegetables and salads		0.50	
Nuts		0.43	
Beans		0.40	
Soy protein		0.28	
Garlic		0.37	
Hydrogenated vegetable oils			0.26
High fat milk			0.39
High fat yogurt			0.51
Red Meat			0.50
Poultry			0.39
Bread			0.42
Rice			0.46
Potato			0.42
Fried foods			0.38
% of variance explained	7.8%	6.3	6.1

The highest factor loading across the factors was reported for each food item. Moreover, assignment of food items to each factor was done based on highest factor loading

Table 4 summarizes the distribution of socio-demographic, lifestyle and job-related variables of study participants across tertiles of dietary patterns. People with higher adherence to Western dietary pattern were significantly more likely to be single (5% vs.2.4%), younger (34.69 ± 6.90 vs. 38.70 ± 7.41), cigarette smokers (11.3% vs.7.9%), and also had a second job (3.7% vs. 2.6%) (*p* < 0.05) and higher mean BMI (26.28 ± 3.78 vs. 24.76 ± 3.64) as compared with those in lower tertiles (*p* < 0.001). The participants in the upper tertile of healthy dietary pattern were predominantly male (31.1% vs. 30%), older (37.18 ± 7.17 vs. 36.32 ± 7.47), with rotational shift works (19.8% vs.16.2%), and without a second job, and also had a higher educational attainment (6–12 years) (21.8% vs.19.5%) (*p* < 0.05). Also, according to our results, subjects in the upper tertile of the traditional dietary pattern were significantly smoker (10.6% vs.9.6%), and rotational shift worker (19.4% vs. 16.9%), and also higher mean BMI (34.91 ± 3.68 vs. 26.14 ± 3.69), higher sleep duration (7.20 ± 1.17 vs. 7.02 ± 1.16)and different distribution of income level (*p* < 0.05).

**Table 4:** Distribution of participant characteristics by tertiles of dietary pattern scores in a large sample of Iranian manufacturing employees

	Western dietary pattern				Healthy dietary pattern				Traditional dietary pattern			
	Tertile 1	Tertile 2	Tertile 3	*P*-value	Tertile 1	Tertile 2	Tertile 3	*P*-value	Tertile 1	Tertile 2	Tertile 3	*P*-value
Age (years)	38.70 ± 7.41	36.77 ± 6.98	34.69 ± 6.90	< 0.001	36.32 ± 7.47	36.64 ± 7.19	37.18 ± 7.17	0.03	36.89 ± 7.23	36.78 ± 7.38	36.49 ± 7.24	0.46
Sex				0.31				0.03				0.21
Male	906 (30.2)	917 (30.6)	925 (30.8)		900 (30.0)	915(30.5)	933 (31.1)		896(29.6)	924(30.8)	928 (30.9)	
female	94 (3.1)	84 (2.8)	75 (2.5)		100 (3.3)	86 (2.9)	67 (2.2)		104(3.5)	77 (2.6)	72(2.4)	
Marital status				< 0.001				0.05				0.32
Married	927(30.9)	927 (30.9)	851 (28.4)		883 (29.4)	907(30.2)	915 (30.5)		892 (29.7)	901 (30.0)	912 (30.4)	
Single	73 (2.4)	74 (2.5)	149 (5.0)		117 (3.9)	94 (3.1)	85 (2.8)		108 (3.6)	100 (3.3)	88 (2,9)	
Education level				0.16				0.004				0.1
0–5 years	95 (3.2)	90 (3.0)	66 (2.2)		95 (3.2)	69(2.3)	87 (2.9)		76 (2.5)	86 (2.9)	89 (3.0)	
6–12	621 (20.7)	613 (20.4)	634 (21.1)		584(19.5)	630(21.0)	654(21.8)		609 (20.3)	611 (20.4)	648 (21.6)	
> 12	284 (9.5)	298 (9.9)	300 (10.0)		321(10.7)	302(10.1)	259(8.6)		315 (10.5)	304 (10.1)	263 (8.8)	
Smoking status				< 0.001				0.76				0.03
Non smokers	762(25.4)	706 (23.5)	661 (22.0)		715 (23.8)	713(23.8)	701 (23.4)		713 (23.8)	735 (24.5)	681 (22.7)	
Current or former smoker	238(7.9)	295 (9.8)	339 (11.3)		285 (9.5)	288(9.6)	299 (10.0)		287 (9.6)	266 (8.9)	319 (10.6)	
BMI (kg/m2)	24.76 ± 3.64	25.64 ± 3.76	26.28 ± 3.78	< 0.001	25.40 ± 3.76	25.75 ± 3.82	25.52 ± 3.75	0.11	26.14 ± 3.69	25.63 ± 3.87	34.91 ± 3.68	< 0.001
Sleep duration(hours)	7.10 ± 1.13	7.15 ± 1.17	7.10 ± 1.18	0.62	7.07 ± 1.18	7.11 ± 1.10	7.17 ± 1.20	0.20	7.02 ± 1.16	7.13 ± 1.15	7.20 ± 1.17	0.002
Physical activity (METs hour /week)	7.24 ± 3.69	7.38 ± 3.55	7.39 ± 3.70	0.61	7.19 ± 3.50	7.30 ± 3.66	7.54 ± 3.77	0.1	7.28 ± 3.74	7.27 ± 3.46	7.46 ± 3.74	0.46
Shift work				0.49				< 0.001				0.02
Daily (daily shift)	450 (15.0)	463(15.4)	436 (14.5)		513(17.1)	430(14.3)	406(13.5)		493 (16.4)	438 (14.6)	418 (13.9)	
Shift (rotating shift work)	550 (18.3)	538(17.9)	564 (18.8)		487 (16.2)	571(19.0)	594(19.8)		507 (16.9)	563 (18.8)	582 (19.4)	
Second job				0.04				0.02				0.18
Yes	79 (2.6)	88 (2.9)	111 (3.7)		74 (2.5)	110(3.7)	94 (3.1)		80 (2.7)	94 (3.1)	104 (3.5)	
No	921(30.7)	913(30.4)	889 (29.6)		926(30.9)	891 (29.7)	906 (30.2)		920 (30.7)	907 (30.2)	896 (29.9)	
Income (Rial)				0.62				0.07				0.001
< 5,000,000	156 (5.2)	157(5.2)	159(5.3)		180 (6.0)	145(4.8)	147(4.9)		129(4.3)	163(5.4)	180(6.0)	
5,000,000–8000000	500 (16.7)	517(17.2)	540 (18.0)		501(16.7)	535(17.8)	521 (17.4)		528(17.6)	522(17.4)	507(16.9)	
8,000,000- 12,000,000	234 (7.8)	211(7.0)	200(6.7)		220 (7.3)	219(7.3)	206 (6.9)		201(6.7)	218(7.3)	226(7.5)	
> 12,000,000	106 (3.5)	114(3.8)	98(3.3)		94 (3.1)	99(3.3)	1(0.0)		137(4.6)	95(3.2)	86(2.9)	
No answer	4(0.1)	2 (0.1)	3 (0.1)		5(0.2)	3(0.1)			5(0.2)	3(0.1)	1(0.0)	

Values are mean (SD) or number (%)

^*^P-values resulted from Chi-squared test and Analysis of variance for categorical and continuous variables, respectively

Table 5 reports the odds ratios of the association between dietary patterns andQoL. Healthy and traditional dietary patterns were significantly associated with quality of life. Our results showed that lower adherence to healthy dietary pattern increased the risk of lowerQoL. Subjects in the lowest tertile of healthy dietary pattern had higher odds of being in low QoL class in the crude model (OR:1.40, 95% CI:1.12–1.74). Also, after adjusting for confounding factors, subjects who were in the first tertile of healthy dietary pattern had 51% higher risk of being in low QoL class (AOR:1.51, 95% CI:1.19–1.91).

**Table 5:** Crude and multivariable adjusted odds ratio (OR) and 95% confidence interval (95% CI for OR) of the association of dietary patterns and QoL in a sample of Iranian manufacturing employees

	Western					Healthy					Traditional				
	Tertile 1^a^		Tertile 2^a^		P trend	Tertile 1^a^		Tertile 2^a^		P trend	Tertile 1^a^		Tertile 2^a^		P trend
	OR	95% CI	OR	95% CI		OR	95% CI	OR	95% CI		OR	95% CI	OR	95% CI	
Crude model	1.01	0.81- 1.25	1.02	0.82- 1.27	0.976	1.40	1.12- 1.74	1.16	0.93- 1.45	0.011	0.78	0.62- 0.10	0.98	0.79- 1.21	0.048
Model 1^*^	0.83	0.66- 1.04	0.93	0.74- 1.16	0.267	1.42	1.14- 1.78	1.18	0.94- 1.48	0.007	0.74	0.60- 0.93	0.96	0.78- 1.20	0.020
Model 2^**^	0.83	0.66- 1.04	0.93	0.74- 1.16	0.270	1.51	1.19- 1.91	1.23	0.97- 1.56	0.003	0.70	0.55- 0.88	0.97	0.77- 1.22	0.004

^*^Model 1 adjusted for age, sex

^**^Model 2 adjusted for age, sex, shifting work, smoking status, BMI, and sleep duration as confounders

^a^In each model the highest (third tertile) was set as reference category

Also, we observed a significant association between traditional dietary patterns and QoL in which lower adherence to this type of dietary pattern decreased the odds of having low QoL. Subjects in the lowest tertile of traditional dietary pattern had lower risk of belonging to the low QoL class in both crude and adjusted models (OR 0.78, 95% CI:0.62–0.97 and AOR 0.70, 95% CI:0.55–0.88, respectively).

Our results showed that the higher intake of western dietary pattern increased risk of low QoL, butthe association was not statistically significant.

We also evaluated the correlation between score of each dietary pattern and VAS score of QoL. Our results showed a significant negative (*r* = -0.093, *P* < 0.001) or positive (*r* = 0.056; *P* = 0.002) correlation between healthy and traditional dietary patterns and QoL, respectively. No significant correlation was observed between western dietary pattern and QoL (*r* = 0.023, *P* = 0.21). Linear regression analysis in unadjusted model showed a nonsignificant association between western dietary pattern and VAS score of QoL (regression coefficient (standard error (SE)) B = 0.0240(0.019), *P* = 0.21); However, after the adjustment for protentional confounder a significant positive association was detected (adjusted regression coefficient B(SE) = 0.049(0.02), *P* = 0.015).A significant negative regression coefficient was observed between healthy dietary pattern and VAS score of QoL (B(SE) = -0.104 (0.02); *P* < 0.001). After the adjustment for confounders, it was remained significant (B(SE) = -0.102(0.02), *P* < 0.001). The evaluation of the association between traditional dietary pattern and VAS score of QoL by linear regression in unadjusted model showed a significant positive relationship (B(SE) = 0.064 (0.021), *P* = 0.002) in adjusted model (B(SE) = 0.096(0.021), *P* < 0.001).

The mean score of each dietary pattern was compared between two extracted classes of QoL as well as between two categories (no problem and some or severe problems) of 5 dimensions of EQ-5D. The results are presented in Supplementary Fig. 1 and Supplementary Fig. 2 (panels A-E). The mean score of healthy and traditional dietary patterns is significantly lower and higher, respectively, in people in low QoL class compared with people in high QoL class (Supplementary Fig. 1, *P* < 0.001). People with mobility problem had significant lower mean score of healthy dietary pattern (Supplementary Fig. 2, panel A), those with pain/discomfort problem had significant higher mean score of traditional dietary pattern and lower mean score of healthy dietary pattern (Supplementary Fig. 2, panel D) and people with depression/anxiety problem had significant higher and lower, respectively, mean scores of traditional and healthy dietary patterns (Supplementary Fig. 2, panel E) as compared with those without the aforementioned problems (all *P* < 0.05).

## Discussion

In this cross-sectional study, we examined the association of major dietary patterns with QoL in a large sample of industrial workers in a developing country. Our results showed that individuals in lower tertiles of healthy dietary pattern were more likely to be in the low QoL class, whilst those in lower tertiles of traditional dietary pattern had a lower risk of being in low QoL class.Furthermore, in this study, there was no significant association between western dietary pattern and QoL.

Data regarding the association between dietary patterns and QoL, not only in the general population, but also in the working population, including industrial workers, are scarce. Choi, et al. reported that lower intakes of fruits and vegetables, which are part of a healthy diet were associated with poor health- related quality of life among operating engineers [4]. In addition, Cash, et al. noted that fast food consumption was associated with reduced productivity in industrial workers [30]. On the other hand, Fitzgerald, et al. showed that having a healthy diet, as measured by adherence to dietary approaches to stop hypertension (DASH), was negatively related to absenteeism in a working population [31]. Rather than specifically related to working society, a systematic review reported that a healthy dietary pattern and Mediterranean dietary pattern were associated with a better dimension of health-related quality of life in both patients and healthy people [32]. Accordingly, due to the impact of QoL on work productivity, the assessment of factors contributing to QoL in industrial workers, including diet, is important.

In line with our study, common characteristics of a healthy dietary pattern and Mediterranean dietary patterns consist of higher amounts of fruits, vegetables, low fat dairies, nuts, fish, and plant base proteins and lower loads of processed meat, red meat, and carbohydrates. Vegetables, fruits, and nuts are rich sources of antioxidants, vitamins and minerals, including C, E and B vitamins. These nutrients enhance mental/mood and physical performance and have protective effects against chronic diseases, psychological disorders, and generally on health and well-being. Plant -based foods reduce cholesterol concentration, blood pressure, and inflammation, resulting in both the enhancement of endothelial function and the regulation of the immune system [33]. Antioxidants reduce the oxidative stress and amount of DNA damage, neuronal cell death, and the accumulation of β-amyloid in the brain [34]. Dairy proteins contain essential amino acid leucine, which could trigger protein synthesis and improvement of muscle mass as well as its function [35] that may affect impact on mobility and self-care. Additionally, low-fat dairy intake could be associated with lower odds of frailty, and have been reported to reduce the inflammatory markers and increase insulin sensitivity [36]. Fish is a major source of omega-3 fatty acids, which possess anti-inflammatory and vascular features that can enhance mental health and reduce the risk of chronic diseases [37, 38]. Considering that QoL is comprised of physical, mental, and motor function of human body, it is of importance that an unhealthy diet has been shown to lead to a reduction in physiological function and an increase in the risk of chronic physical diseases development through affecting immune and cognitive functions [32] and, consequently, an improvement in diet represents a key factor for improvement of QoL.

The traditional dietary pattern of Iranians is high in hydrogenated oils, red meat, bread, rice, potato and fried foods. High saturated and trans-fat, refined sugars and red meat can decrease the brain derived neurotropic factor (BDNF) concentrations and deteriorate mental health, which can affect QoL [39]. These food groups are also shown to increase insulin resistance and inflammatory markers concentration, including interleukin-6 and C reactive protein, which can result in a higher risk of chronic disease and psychiatric disorders [37, 40].Therefore, it is plausible that these could have detrimental impacts on QoL.

Although the association of western dietary pattern with QoL was not statistically significant in our study, previous studies showed that the western dietary pattern might be associated with lower QoL in all domains [41, 42]. Several studies have suggested that the adherence to western dietary pattern may be associated with an increased risk of anxiety, depression, and mental symptoms, all of which can have impact on QoL [43]. Additionally, western diet and Iranian traditional foods share some food items, such as red meat, refined grain, and hydrogenated oils, so the mechanism of action of western dietary pattern on quality of life is likely comparable to that of the traditional dietary pattern. The lack of significant negative association between western dietary pattern and QoL, in spite of similarity with Iranian traditional dietary pattern, might be due to this reason that saturated and trans fats, refined grains, rice and fried foods (nutrients and food groups considered responsible for declining in QoL) had higher factor loadings in traditional dietary pattern in our study population compared to western dietary pattern.

Industrial employees are especially prone to low QoL, due to the long hours of work, fatigue, irregular sleep hours, and low income level in many of them. Healthy dietary patterns and healthy food choices corresponding to their situation and income level can be a helpful approach to improve their QoL by reducing the risk of chronic diseases and improving their mental status (37). Among variables confounding the association betwen dietary pattern and QoL, BMI and education level of people could be particularly important. Increased BMI could affect mobility, leading to osteoarthritis. Furthermore, higher BMI is associated with increased risk of chronic diseases, which can have a detrimental impact on QoL. [44, 45]. The findings of this study showed that BMI increased across the tertiles of traditional dietary pattern, whilst, as mentioned above, lower tertiles of this pattern were related to better QoL.Therefore, it seems that adherence to the Iranian traditional diet, as the major dietary pattern, may result in higher BMI and lower QoL. Moreover, our results indicated that subjects in the first tertile of traditional pattern were more likely to undertake daily shift work rather than night shifts. This result is concordant with previous studies suggesting that night shift work can affect eating habits, leading to unhealthy diets [46]. In addition, it is believed that socioeconomic status can have an impact on dietary habits and well-being [40]; indeed, the findings of our study confirm this, by showing that people in higher tertiles of healthy dietary pattern are more likely to have higher education levels. Also, several studies have revealed that higher education level is related to higher QoL[47].

However, this study has some limitations that should be addressed. First, we could not derive any cause-effect relationship from the findings of this study due to the cross-sectional design. Second, some subjective decisions were taken in factor analysis and latent class analysis regarding the extraction of dietary patterns and class of QoL, respectively, such as the number of factors and classes to be extracted and labeling of factors/ classes and interpreting the results. Third, self-report questionnaires were used to gather data in this study, which could lead to misclassification of participants and recall bias. Finally, the effect of ‘healthy worker’ could have occurred in this study. Despite the aforementioned limitations, this study has some strengths that should be highlighted. To our knowledge, this is the first study with alarge sample size that evaluated the association between dietary patterns and QoL among industrial employees using a comprehensive and advanced statistical method. Moreover, the impact of a wide variety of potential confounders on results has been adjusted in this study.

## Conclusions

The results of this study showed that adhering to a healthy diet was associated with better QoLin manufacturing employees. Also, lower adherence to the traditional dietary pattern of Iranian society was related to higher QoL. Health policy makers could use these findings to develop policies in order to improve dietary quality of employees, such as through education programs and considering accommodations for healthy dietary products. Furthermore, intervention studies are needed to investigate the effects sof different healthy dietary patterns, such as DASH and Mediterranean diet, on quality of life in manufacturing employees.

### Supplementary Information

**Additional file 1.** Mean score (95%CI for mean) of each dietary pattern in two classes of QoL.

**Additional file 2.** Mean score (95%CI for mean) of each dietary pattern in two categories of mobility problem (A), self-care problem (B), daily activity problem (C), pain/discomfort problem (D) and depression/anxiety problem (E) as items of QoL.

## Data Availability

The data are available from corresponding author upon justifiable request.

## Abbreviations

WHO: World Health Organization
QoL: Quality of life
BMI: Body mass index
PCA: Principal component analysis
ESCO: Esfahan Steel Company
FFQ: Food frequency questionnaire
VAS: Visual analog scale
IPAQ: The International Physical Activity Questionnaire
EFA: Exploratory Factor Analysis
LCA: Latent class analysis
ANOVA: One-way analysis of variance
DASH: Dietary approaches to stop hypertension
BDNF: Brain derived neurotropic factor
