# Supplementary figures and images for: The association between dietary patterns and quality of life: a cross-sectional study among a large sample of industrial employees
Source: BMC Public Health. 2023 Oct 17;23:2016. doi: 10.1186/s12889-023-16898-9 (PMC10580545; doi:10.1186/s12889-023-16898-9)

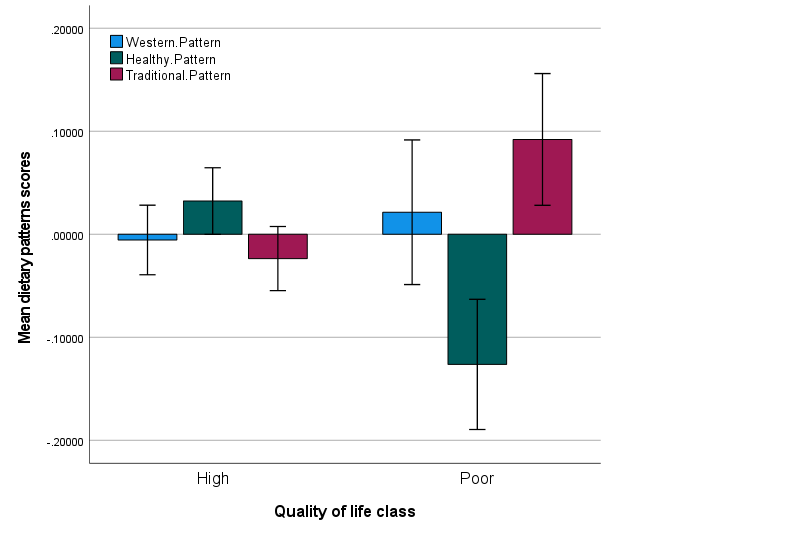


Figure 1: Mean score (95%CI for mean) of each dietary pattern in two classes of QoL

Supplement: Supplementary file 1 — Additional file 1. Mean score (95%CI for mean) of each dietary pattern in two classes of QoL. [file 12889_2023_16898_MOESM1_ESM.docx]
