# Supplementary material for: The association between dietary patterns and quality of life: a cross-sectional study among a large sample of industrial employees
Source: BMC Public Health. 2023 Oct 17;23:2016. doi: 10.1186/s12889-023-16898-9 (PMC10580545; doi:10.1186/s12889-023-16898-9)
Supplement: Supplementary file 2 — Additional file 2. Mean score (95%CI for mean) of each dietary pattern in two categories of mobility problem (A), self-care problem (B), daily activity problem (C), pain/discomfort problem (D) and depression/anxiety problem (E) as items of QoL. [file 12889_2023_16898_MOESM2_ESM.docx]

| 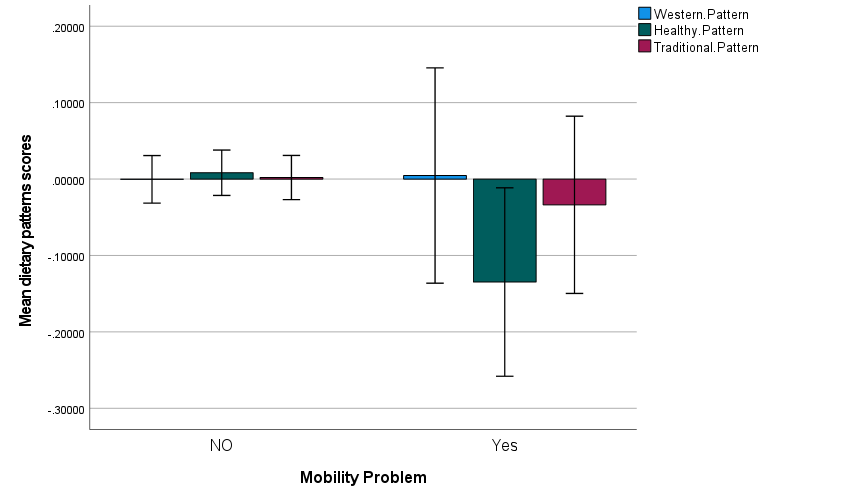  A | 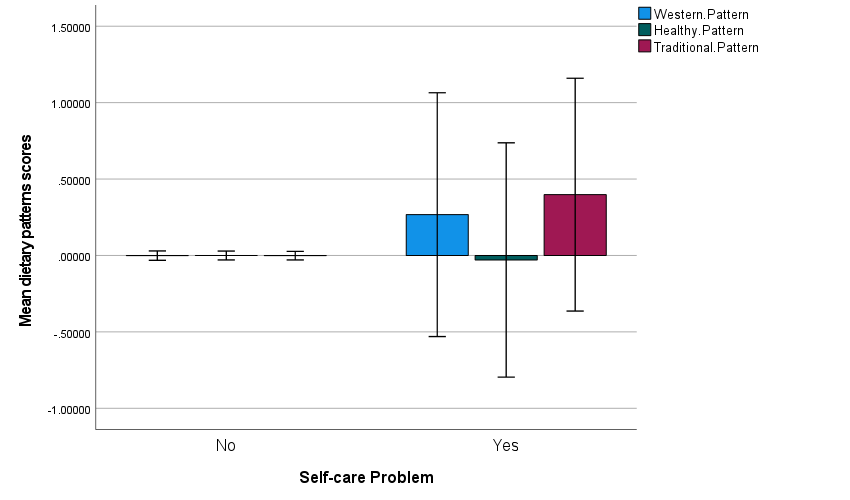  B |
| --- | --- |
| 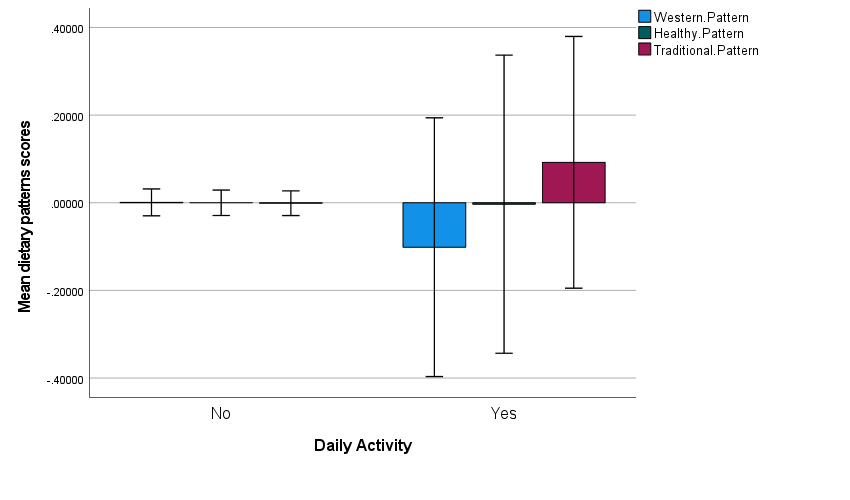  C | 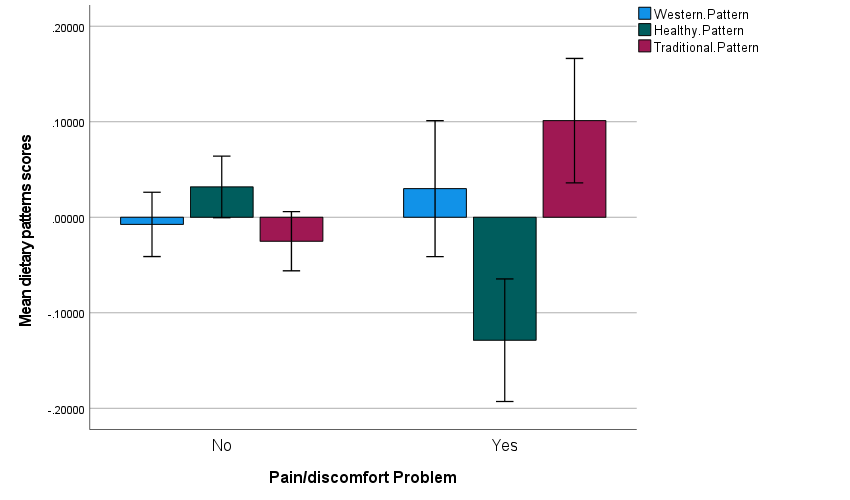  D |
| 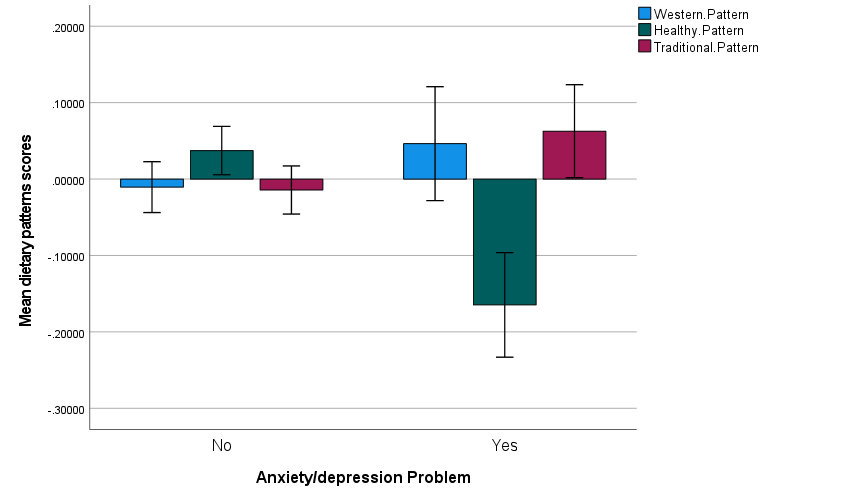  E | |

Figure 2: Mean score (95%CI for mean) of each dietary pattern in two categories of mobility problem (A) , self-care problem (B), daily activity problem (C), pain/discomfort problem (D) and depression/anxiety problem (E) as items of QoL
